# Associations of Biomarkers of Kidney Tubule Health with Retinal Microvascular Signs: The Multi-Ethnic Study of Atherosclerosis

**DOI:** 10.34067/KID.0000000970

**Published:** 2025-09-05

**Authors:** Armin Ahmadi, Hassan Gorji, Sanskrita Shashikant, Ronit Katz, Alfons J.H.M. Houben, Robert N. Weinreb, Matthew Allison, Stacy M. Meuer, Barbara E. Klein, Mary Frances Cotch, Orlando M. Gutierrez, Mark J. Sarnak, Michael Shlipak, Joachim H. Ix, Rakesh Malhotra

**Affiliations:** 1 Division of Nephrology-Hypertension, Department of Medicine, University of California San Diego, San Diego, California; 2 Department of Obstetrics and Gynecology, University of Washington, Seattle, Washington; 3 Department of Internal Medicine and Cardiovascular Research Institute Maastricht, Maastricht University Medical Center, Maastricht, The Netherlands; 4 Department of Ophthalmology, University of California San Diego, San Diego, California; 5 Department of Family Medicine, University of California San Diego, San Diego, California; 6 Department of Ophthalmology and Visual Sciences, University of Wisconsin School of Medicine and Public Health, Madison, Wisconsin; 7 Office of Vision Health and Population Sciences, National Eye Institute, National Institutes of Health, Bethesda, Maryland; 8 Departments of Medicine and Epidemiology, University of Alabama at Birmingham, Birmingham, Alabama; 9 Division of Nephrology, Department of Medicine, Tufts Medical Center, Boston, Massachusetts; 10 Department of Medicine, Kidney Health Research Collaborative, San Francisco VA Healthcare System, University of California, San Francisco, San Francisco, California

**Keywords:** cardiovascular disease, CKD, kidney tubule, vascular disease, biomarkers

## Abstract

**Key Points:**

- Kidney injury molecule-1 and soluble urokinase plasminogen activator receptor are associated with retinal microvascular changes in individuals without CKD, diabetes, or cardiovascular disease.
- Tubule biomarkers may reveal microvascular pathways linking kidney dysfunction to cardiovascular risk beyond eGFR or albumin-to-creatinine ratio.

**Background:**

CKD is strongly associated with cardiovascular disease (CVD), yet the etiology responsible for this link remains elusive. Novel blood and urine biomarkers reflecting kidney tubule dysfunction and injury may provide novel insights to mechanisms linking the kidney to CVD.

**Methods:**

In 470 participants of the Multi-Ethnic Study of Atherosclerosis without type 2 diabetes, CVD, or CKD, we measured six plasma (kidney injury molecule-1 [KIM-1], monocyte chemoattractant protein-1, soluble urokinase plasminogen activator receptor, tumor necrosis factor receptor 1 and 2, and anti–chitinase-3-like protein 1) and six urinary (*α* 1 microglobulin, EGF, KIM-1, monocyte chemoattractant protein-1, anti–chitinase-3-like protein 1, and uromodulin) kidney tubule health biomarkers. To assess microvascular health, we used retinal microvascular measurements assessed from fundus photography: central retinal arteriolar and venular equivalents (central retinal artery equivalent [CRAE] and central retinal venular equivalent [CRVE], respectively). Multivariable linear regression evaluated associations of tubule biomarkers and kidney function with CRAE and CRVE.

**Results:**

The mean participant age was 60±10 years with 52% female. The racial and ethnic distribution was 46% White, 24% Black, 18% Hispanic, and 11% Chinese. The mean eGFR was 92.1±13.3 ml/min per 1.73 m^2^, and the median urine albumin-to-creatinine ratio was 4.7 mg/g (interquartile range, 3.0–9.4). Higher plasma KIM-1 (*β*, −5.14; 95% confidence interval [CI], −9.84 to −0.45) and urine KIM-1 (*β,* −5.68; 95% CI, −10.15 to −1.22) concentrations were individually associated with narrower CRAE, while plasma soluble urokinase plasminogen activator receptor concentrations were individually associated with wider CRAE (*β*, 9.15; 95% CI, 0.89 to 17.4) and CRVE (*β*, 21.49; 95% CI, 9.39 to 33.59). There were no significant associations between the remaining tubule health biomarkers and CRAE or CRVE nor were there associations between eGFR or urine albumin-to-creatinine ratio with CRAE and CRVE.

**Conclusions:**

In this study of community-living individuals without CKD, diabetes, or CVD, selected kidney tubule health markers are associated with retinal microvascular changes. These findings suggest that kidney tubule biomarkers may reflect or contribute to systemic microvascular dysfunction, above and beyond glomerular damage. Tubular biomarkers may help elucidate the shared microvascular mechanisms linking CKD and CVD.

## Visual Abstract

**Figure:**
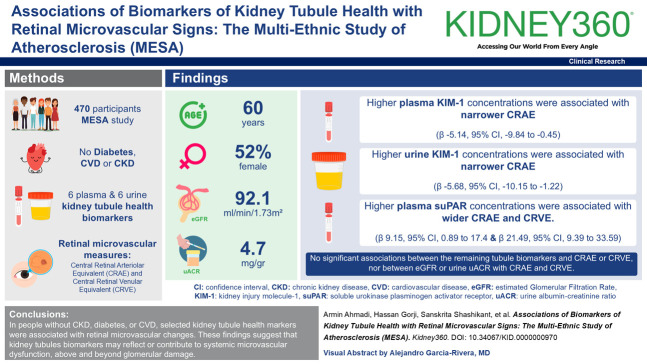

## Introduction

Modest reductions in kidney function, as captured by the glomerular markers eGFR and albuminuria, strongly associate with cardiovascular disease (CVD), but the mechanisms are elusive.^1^ These glomerular markers are widely available clinically but do not capture kidney tubule damage or dysfunction.^2^

In biopsy studies, kidney tubulo-interstitial fibrosis is the strongest predictor of kidney disease progression.^3^ Novel noninvasive biomarkers that give insights to kidney tubule dysfunction and injury have been identified. In our prior work, higher levels of these biomarkers were strongly associated, and in some instances predictive, of CVD events, above and beyond eGFR and albuminuria.^4–6^ Many of these biomarkers are produced within the kidney tissue and eliminated in the urine but provide insights to CVD.^5,6^ We hypothesized that biomarkers of kidney tubule injury may reflect systemic microvascular dysfunction, which may underlie both CVD and progressive kidney damage; however, the association between kidney tubule biomarkers and systemic microvascular dysfunction has not previously been investigated.

Recent advancement in digital retinal imaging has provided a noninvasive diagnostic tool to evaluate microvascular function in the eye.^7,8^ Narrower retinal arteriolar diameter and, conversely, wider venular diameter have been associated with impaired vascular function and increased CVD risk in the Atherosclerosis Risk in Communities Study.^9,10^ In Asian populations, retinal arteriolar narrowing and venular widening have been associated with incident CKD.^11,12^ Given the similarities in arteriolar, capillary, and venular size between the eye and the kidney, biomarkers of tubular injury may offer novel insights into systemic microvascular pathology and its manifestations in the retinal circulation.^7^

The Multi-Ethnic Study of Atherosclerosis (MESA) is a multicenter prospective cohort study conducted among adults without clinically apparent CVD and focused on novel risk factors for developing CVD.^13^ A previous study from MESA that used the full cohort demonstrated that albuminuria, a marker of renal endothelial damage, is associated with narrower retinal artery caliber and wider retinal venular caliber.^13,14^ A separate study evaluated a nested subset of MESA participants without diabetes or CKD, measured tubule health markers, and demonstrated that several were associated with incident CKD development during follow-up. Using these measures from this tubule health ancillary study, the analysis presented below tests the hypothesis that kidney tubule biomarkers, reflecting tubular injury and dysfunction, are associated with retinal microvascular dysfunction independent of albuminuria, eGFR, and established risk factors for microvascular disease.

## Methods

### Study Population

**Figure 1:**
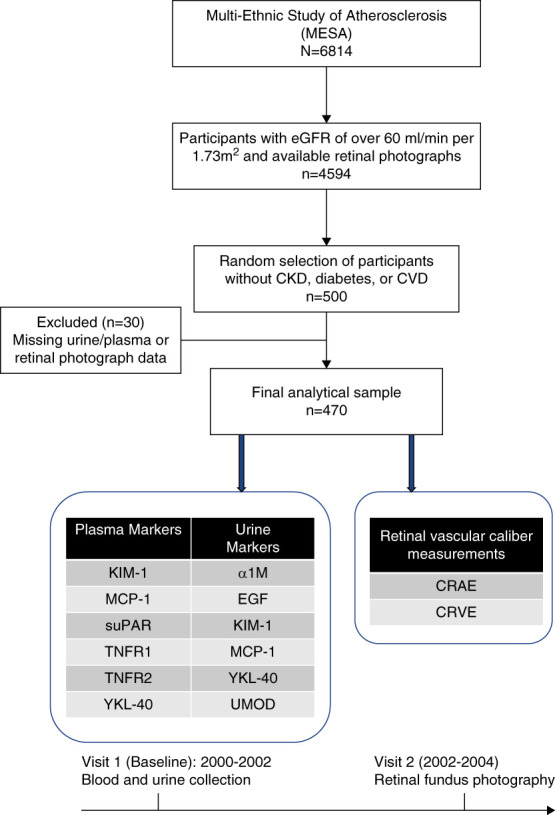
**Flow chart of the study.** *α*1M, *α* 1 microglobulin; CRAE, central retinal arteriolar equivalent, CRVE, central retinal venular equivalent; CVD, cardiovascular disease; KIM-1, kidney injury molecule-1, MCP-1, monocyte chemoattractant protein-1; suPAR, soluble urokinase-type plasminogen activator receptor; TNFR, tumor necrosis factor receptor; UMOD, uromodulin; YKL-40, anti–chitinase-3-like protein 1.

MESA recruited 6814 community-living individuals between July 2000 and July 2002. A detailed description of study participants and methods has been published previously.^15^ Prevalent clinically apparent CVD was exclusion criteria. Among participants, 67% (4594) had an eGFR of ≥60 ml/min per 1.73 m^2^ on the basis of the CKD-Epidemiology Collaboration equation at baseline (visit 1; July 2000–July 2002) with available retinal photographs collected at visit 2 (August 2002–January 2004). Kidney tubule measurements for this study used a previous ancillary study designed to evaluate incident CKD and included 500 individuals who did not have diabetes or CKD (defined as eGFR <60 ml/min per 1.73 m^2^) at baseline. Diabetes mellitus was defined as being present if the fasting glucose was ≥126 mg/dl or use of insulin or oral hypoglycemic medication. CVD was defined by self-reported history or clinical evidence of myocardial infarction, angina, heart failure, stroke, transient ischemic attack, or prior coronary revascularization procedures. Of these, 30 were missing urine samples for biomarker measurements or retinal photographs, yielding a sample of 470 participants, all of whom were without CVD, CKD, or diabetes available for this analysis (Figure 1). Although albuminuria was not used as an exclusion criteria, the median (interquartile range [IQR]) urine albumin-to-creatinine ratio in the final analytic sample was 4.7 mg/g (3.0–9.4), consistent with preserved kidney function and minimal proteinuria.

### Kidney Tubular Health Biomarkers

Six plasma (kidney injury molecule-1 [KIM-1], monocyte chemoattractant protein-1 [MCP-1], soluble urokinase plasminogen activator receptor [suPAR], tumor necrosis factor receptor [TNFR] 1 and 2, and anti–chitinase-3-like protein 1 [YKL-40]) and six urinary (*α* 1 microglobulin [*α*1M], EGF, KIM-1, MCP-1, YKL-40, and uromodulin) kidney tubule health biomarkers were measured using samples stored at −80°C after a single freeze-thaw. All marker measurements were performed in the CKD Biomarkers Consortium Central Laboratory at Brigham and Women's Hospital. These samples were collected at the baseline MESA examination. Biomarkers were selected based on previous studies showing association with kidney tubular biology including tubular injury, inflammation, and repair.^16,17^ The plasma biomarkers were measured using a multiplex assay (Meso Scale Diagnostics, Rockville, MD). The urine biomarkers were measured using a multiplex assay (Luminex, Austin, TX) except for A1M, which was measured by nephelometry. Biomarkers were measured in duplicates, and the results were averaged to improve precision, except for A1M as prior data demonstrated very low CVs for this analyte. Urine albumin concentration was measured by the BNII ProSpec nephelometer (Siemens AG, Munich, Germany), and urine creatinine concentration was measured by the rate Jaffé method (Roche/Hitachi, Basel, Switzerland). The intra-assay and interassay coefficients of variation for markers were all <10%.

### Retinal Photography and Retinal Microvascular Caliber Measurements

Central retinal artery equivalent (CRAE) and central retinal venular equivalent (CRVE) measurements were made at MESA visit 2 (2002–2004) using a standardized protocol.^18^ A detailed description of retinal measurements has been described previously.^19,20^ In brief, retinal images were obtained without pharmacologic pupil dilation using a 45-degree 6.3-megapixel digital nonmydriatic camera. The obtained photographed fields for each eye were sent to the Ocular Epidemiology Reading Center at University of Wisconsin-Madison for retinal vascular caliber measurements.^20^ All photographs were scored by trained graders blinded to patient's characteristics. Retinal microvascular caliber was measured as a quantification of generalized vessel narrowing using a semiautomated computer-assisted program (IVAN, University of Wisconsin, Madison).^21^ For each photograph, all arterioles and venules coursing through an area 0.5–1 disc diameter from the optic disc margin were measured and the biggest six arteriolar and venular calibers were summarized as the CRAE and CRVE.^22,23^

### Statistical Analysis

Continuous variables are described as the mean (SD) or median and IQRs and categorical variables as absolute (*n*) and relative (%) frequency. To test the cross-sectional associations between plasma (KIM-1, MCP-1, suPAR, TNFR1, TNFR2, and YKL-40) and urine (*α*-1M, EGF, KIM-1, MCP-1, YKL-40, and uromodulin) biomarkers with CRAE and CRVE, we used multivariable linear regression through a series of nested models. Since most of the biomarkers were right-skewed, we normalized the data by applying a log transformation to the individual biomarker concentrations in all regression analyses. Consequently, a 1% change in each biomarker is associated with a *β*/100 change in the outcome variables (CRAE and CRVE). The first model was unadjusted. The final model was adjusted for age, sex, race and ethnicity, urine creatinine (to account for urine tonicity at the time of urine collection), smoking status, hypertension (defined as a systolic BP ≥140 mm Hg, diastolic BP ≥90 mm Hg, or use of medication prescribed for hypertension), body mass index, low-density lipoprotein cholesterol, high-density lipoprotein cholesterol, eGFR, and urine albumin. We fit separate sets of models for CRAE and CRVE. All analyses were performed using SPSS and Stata 11. *P* values <0.05 were considered statistically significant in all models.

## Results

Table 1 presents baseline characteristics of the study population. Among the 470 participants, the mean age was 60±10 years, 52% were female, and 36% had hypertension. Forty-six percent were non-Hispanic White, 24% were Black, 18% were Hispanic, and 11% were Chinese. By design, none of the participants had CVD, diabetes, or CKD. The mean eGFR was 92±13 ml/min per 1.73 m^2^, and the median urine albumin-to-creatinine ratio was 4.7 mg/g (IQR, 3.0–9.4). The mean (SD) baseline value of CRAE was 144 (14) *μ*m and CRVE was 214 (21) *μ*m. Supplemental Table 1 presents the median and IQR along with log transformed mean values for the measured plasma and urinary biomarkers.

**Table 1:** Characteristics of the analyzed population

Characteristics	Study Population *n*=5470
Age (yr), mean±SD	60±10
**Sex, *n* (%)**	
Male	225 (48)
Female	245 (52)
**Race, *n* (%)**	
Black	112 (24)
Chinese	53 (11)
Hispanic	87 (19)
White	218 (46)
BMI (Kg/m^2^), mean±SD	28±5
Hypertension, *n* (%)	169 (36)
Systolic BP (mm Hg), mean±SD	123±20
Diastolic BP (mm Hg), mean±SD	72±10
**Smoking, *n* (%)**	
Never	227 (48)
Former	179 (38)
Current	64 (14)
Total cholesterol (mg/dl), mean±SD	193±37
LDL (mg/dl), mean±SD	117±33
HDL (mg/dl), mean±SD	51±15
Triglycerides (mg/dl)	129±75
Urine albumin/creatinine (mg/g), median (IQR)	4.7 (3-9.4)
eGFR (ml/min per 1.73 m^2^), mean±SD	92±13
CRAE (*μ*m), mean±SD	144±14
CRVE (*μ*m), mean±SD	214±21

Data are means (SDs) for continuous variables and *N* (percentages) for categorical variables. BMI, body mass index; CRAE, central retinal arteriolar equivalent; CRVE, central retinal venular equivalent; HDL, high-density lipoprotein; LDL, low-density lipoprotein.

**Figure 2:**
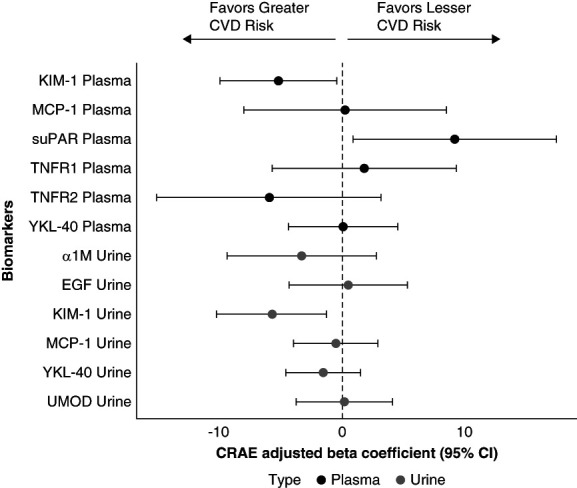
**Forest plot of multivariable regression coefficients (95% CI) representing the association between a urine and plasma kidney tubule health biomarkers and CRAE.** The coefficients were scaled to reflect a 1% change in the biomarker. Thus, a 1% increase in the biomarker corresponds to a *β*/100 change in the CRAE. CI, confidence interval; CVD, cardiovascular disease.

In the unadjusted model, higher plasma KIM-1 and TNRF2 and urine KIM-1 and A1M concentrations were individually associated with narrower CRAE (Table 2). In the fully adjusted model, higher plasma KIM-1 (*β*, −5.14; 95% confidence interval [CI], −9.84 to −0.45; *P* = 0.03), and urine KIM-1 (*β*, −5.68; 95% CI, −10.15 to −1.22; *P* = 0.01) concentrations remained associated with narrower CRAE (Figure 2 and Table 2). In addition, higher plasma suPAR concentrations were associated with wider CRAE (*β*, 9.15; 95% CI, 0.89 to 17.4; *P* = 0.03; Figure 2 and Table 2).

**Table 2:** The association of plasma kidney tubule health biomarkers with central retinal arteriolar equivalent (*n*=470) in persons without CKD, cardiovascular disease, or diabetes

Plasma Markers CRAE	Model 1 *β*, 95% CI	Model 1 *P* Value	Final Model *β*, 95% CI	Final Model *P* Value
KIM-1 (pg/ml)	−6.94 (−11.60 to −2.29)^a^	0.004^a^	−5.14 (−9.84 to −0.45)^a^	0.03^a^
MCP-1 (pg/ml)	−2.52 (−10.94 to 5.89)	0.56	0.28 (−8.01 to 8.56)	0.95
suPAR (pg/ml)	6.99 (−1.22 to 15.26)	0.10	9.15 (0.89 to 17.40)^a^	0.03^a^
TNFR1 (pg/ml)	−3.39 (−10.76 to 3.98)	0.37	1.80 (−5.69 to 9.29)	0.64
TNFR2 (pg/ml)	−9.53 (−18.69 to −0.37)^a^	0.04^a^	−5.90 (−15.01 to 3.20)	0.20
YKL-40 (pg/ml)	−3.62 (−7.71 to 0.47)	0.08	0.09 (−4.34 to 4.53)	0.97
**Urine markers CRAE**				
*α*1M (mg/L)	−6.96 (−12.95 to −0.97)^a^	0.02^a^	−3.26 (−9.32 to 2.80)	0.29
EGF (pg/ml)	3.016 (−1.80 to 7.83)	0.22	0.49 (−4.31 to 5.28)	0.84
KIM-1 (pg/ml)	−3.63 (−7.02 to −0.247)^a^	0.04^a^	−5.68 (−10.15 to −1.22)^a^	0.01^a^
MCP-1 (pg/ml)	−2.21 (−5.68 to 1.26)	0.21	−0.51 (−3.94 to 2.93)	0.77
YKL-40 (pg/ml)	−1.63 (−4.70 to 1.44)	0.30	−1.52 (−4.55 to 1.52)	0.33
UMOD (pg/ml)	1.43 (−2.55 to 5.40)	0.48	0.19 (−3.74 to 4.12)	0.93
**Glomerular markers CRAE**				
eGFR (ml/min per 1.73 m^2^)	0.09 (0.01 to 0.17)^a^	0.03	0.006 (−0.09 to 0.10)	0.91
Urine ACR mg/g	−0.07 (−0.13 to −0.01)^a^	0.02	−0.05 (−0.11 to 0.01)	0.13

A 1% increase in the biomarker corresponds to a *β*/100 change in CRAE and CRVE.

Model 1 is biomarker alone. The final model is adjusted for age, race and ethnicity, sex, body mass index, hypertension, low-density lipoprotein, high-density lipoprotein, smoking status, urine albumin, urine creatinine, and eGFR.

ACR, albumin-to-creatinine ratio; *α*1M, *α* 1 microglobulin; CI, confidence interval; CRAE, central retinal arteriolar equivalent; CRVE, central retinal venular equivalent; CVD, cardiovascular disease; KIM-1, kidney injury molecule-1, MCP-1, monocyte chemoattractant protein-1; suPAR, soluble urokinase-type plasminogen activator receptor; TNFR, tumor necrosis factor receptor; UMOD, uromodulin; YKL-40, anti–chitinase-3-like protein 1.

a The associations with *P* < 0.05.

**Figure 3:**
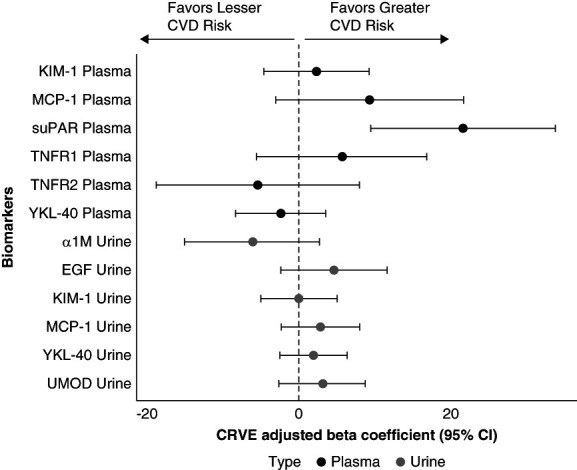
**Forest plot of multivariable regression coefficients (95% CI) representing the association between a urine and plasma kidney tubule health biomarkers and CRVE.** The coefficients were scaled to reflect a 1% change in the biomarker. Thus, a 1% increase in the biomarker corresponds to a *β*/100 change in the CRVE.

For CRVE, higher plasma suPAR concentrations were associated with wider CRVE in both unadjusted and fully adjusted models (*β*, 21.5; 95% CI, 9.4 to 33.6; *P* = 0.001; Figure 3 and Table 3). Although higher urine EGF concentrations were associated with wider CRVE in the unadjusted model, these associations were attenuated in the final model (Table 3). There were no significant associations between the remaining biomarkers with either CRAE or CRVE (Tables 2 and 3).

**Table 3:** The association of urine kidney tubule health biomarkers with central retinal venular equivalent (*n*=470) in persons without CKD, cardiovascular disease, or diabetes

Plasma Markers CRVE	Model 1 *β*, 95% CI	Model 1 *P* value	Final Model *β*, 95% CI	Final Model *P* value
KIM-1 (pg/ml)	−2.32 (−9.25 to 4.62)	0.51	2.30 (−4.65 to 9.25)	0.52
MCP-1 (pg/ml)	9.72 (−2.68 to 22.12)	0.12	9.24 (−3.01 to 21.49)	0.14
suPAR (pg/ml)	19.52 (7.39 to 31.65)^a^	0.002^a^	21.49 (9.39 to 33.59)^a^	0.001^a^
TNFR1 (pg/ml)	−0.13 (−11.02 to 10.77)	0.98	5.58 (−5.56 to 16.73)	0.33
TNFR2 (pg/ml)	−5.38 (−18.90 to 8.20)	0.44	−5.41 (−18.67 to 7.85)	0.42
YKL-40 (pg/ml)	−3.25 (−9.30 to 2.81)	0.29	−2.40 (−8.30 to 3.50)	0.43
**Urine markers CRVE**				
*α*1M (mg/L)	−1.01 (−9.91 to 7.88)	0.82	−6.15 (−15.01 to 2.71)	0.17
EGF (pg/ml)	7.48 (0.39 to 14.57)^a^	0.04^a^	4.59 (−2.41 to 11.59)	0.20
KIM-1 (pg/ml)	0.99 (−4.03 to 6.02)	0.70	−0.02 (−5.03 to 4.99)	0.99
MCP-1 (pg/ml)	4.58 (−0.53 to 9.70)	0.08	2.78 (−2.39 to 7.95)	0.29
YKL-40 (pg/ml)	1.83 (−2.69 to 6.36)	0.73	1.90 (−2.59 to 6.39)	0.41
UMOD (pg/ml)	4.04 (−1.82 to 9.90)	0.18	3.06 (−2.63 to 8.74)	0.29
**Glomerular markers CRVE**				
eGFR (ml/min per 1.73 m^2^)	0.08 (−0.46 to −0.20)	0.22	0.06 (−0.09 to 0.20)	0.45
Urine ACR (mg/g)	−0.02 (−0.11 to 0.07)	0.62	−0.01 (−0.10 to 0.08)	0.82

A 1% increase in the biomarker corresponds to a β/100 change in CRAE and CRVE.

Model 1 is biomarker alone. The final model is adjusted for age, race and ethnicity, sex, body mass index, hypertension, low-density lipoprotein, high-density lipoprotein, smoking status, urine albumin, urine creatinine, and eGFR.

ACR, albumin-to-creatinine ratio; *α*1M, *α* 1 microglobulin; CI, confidence interval; CRAE, central retinal arteriolar equivalent; CRVE, central retinal venular equivalent; KIM-1, kidney injury molecule-1, MCP-1, monocyte chemoattractant protein-1; suPAR, soluble urokinase-type plasminogen activator receptor; TNFR, tumor necrosis factor receptor; UMOD, uromodulin; YKL-40, anti–chitinase-3-like protein 1.

a The associations with *P* < 0.05.

Regarding clinical markers of kidney function, while there were signals with CRAE and CRVE in unadjusted models, these were attenuated and not significant in adjusted models (Tables 2 and 3).

## Discussion

In this study of community-living individuals without CVD, CKD, or diabetes, we observed significant associations between a select set of kidney tubule biomarkers and retinal microvascular measurements. Previous studies of MESA have shown that narrower CRAE or arteriolar narrowing is associated with risk of stroke,^24^ hypertension,^23^ and CKD development^19^ while a wider CRVE or venular widening is linked to hypertension,^25^ dyslipidemia, and systemic inflammation.^23^ We found that higher plasma and urine KIM-1 levels were associated with narrower CRAE indicating a potential shared pathway between kidney tubule injury and retinal microvascular disease, suggesting a link between the kidney tubules and systemic microvascular disease. CRAE and CRVE were not associated with eGFR or albuminuria. This may suggest stronger links with kidney tubule damage and dysfunction than with glomerular disease; however, we selected participants for eGFR ≥60 ml/min per 1.73 m^2^, which may have constrained the range of values for the glomerular markers. Collectively, our findings provide evidence in support of a shared microvascular mechanisms linking kidney tubule dysfunction and injury with systemic microvascular disease.

KIM-1 is a urinary injury marker that is highly upregulated in the proximal tubules in response to kidney injury.^26^ Increased levels of plasma and urine KIM-1 have been shown to be associated with AKI and CKD progression, and KIM-1 has been qualified by the US Food and Drug Administration as a biomarker of kidney injury for drug toxicity studies.^26^ KIM-1 is only produced in the kidney, so both higher plasma and urine levels likely reflect kidney tubule pathology, and it is unlikely that higher plasma levels of KIM-1 would reflect injury in nonkidney tissues. Whether measured in plasma or urine, we find that higher KIM-1 concentrations are associated with narrower CRAE, independent of the glomerular kidney measures (eGFR and albuminuria) and other CVD risk factors. At least two previous studies have reported that urine concentrations of KIM-1 were independently associated with CVD events.^4,27^ Similarly, two previous studies suggest that narrower CRAE is associated with CKD and CVD.^11,23^ Specifically, a prospective study of 1256 individuals from Singapore reported that narrower retinal arterioles and wider retinal venules were associated with increased risk of incident CKD.^11^ Within MESA, narrower retinal arterioles were associated with increased risk of stroke, hypertension, and CKD development.^19,23,26^ Thus, the association of higher KIM-1 with narrower CRAE suggests that systemic microvascular dysfunction may simultaneously affect the retina, kidney tubules, and cardiovascular system and may help explain why previous studies have found associations of kidney tubule health markers in urine are so strongly associated with, and indeed predictive of, CVD events.^5^

Although the link between both plasma and urine KIM-1 associated with narrower CRAE, we found no association of any other plasma or urine biomarker with narrower CRAE. The findings with plasma suPAR are complex, as we found a statistically significant association in the opposite direction—with wider CRAE—suggesting a link in a direction previously lower risk of CVD. This association was unexpected as higher suPAR was also associated with higher retinal venous diameter, which is linked with higher CVD event risk.^23,28^ This observation may, in part, be due to the differential impact of comorbidities such as hypertension and (pre)diabetes or inflammation on CRAE diameter.^14,29^ This is also supported by a previous MESA study which demonstrated a *U*-shaped distribution of albuminuria across a range of CRAE measures with highest prevalence seen in the narrowest and widest CRAE.^14^ Wider retinal venular caliber measures (but not arterioles) have been purported as a marker of systemic inflammation, suggesting that inflammation contributes to microvascular dysfunction through venular changes.^30,31^ suPAR is a well-established biomarker of chronic systemic inflammation and immune activation, and its elevation is thought to reflect upstream pro-inflammatory signaling that adversely affects vascular integrity.^32^ Experimental and clinical studies have shown that suPAR can promote endothelial dysfunction through activation of inflammatory and integrin-mediated pathways.^33–35^ These mechanisms may contribute to narrowing of the arterioles in some vascular beds, while also promoting venular dilation through increased endothelial permeability, vascular remodeling, or inflammatory cell trafficking.^36–38^ Thus, the divergent associations observed in our study (wider CRAE and CRVE with higher suPAR) may reflect complex, vessel-specific responses to inflammation that differ depending on stage of injury, hemodynamic factors, or microvascular territory. Previous studies evaluating associations of suPAR with CVD demonstrate that higher suPAR levels have been linked with CVD events including coronary artery disease, acute coronary syndromes, and heart failure in several cohorts.^39–43^ In a cross-sectional study of patients with non-obstructive coronary artery disease, higher plasma suPAR was identified as an independence predictor of coronary microvascular function suggesting the key role of inflammation and immune dysregulation in driving microvascular dysfunction.^44^ Elevated circulating suPAR levels can potentially drive microvascular dysfunction through several processes including exacerbating inflammatory burden, damaging endothelial cells lining of microvasculature disrupting normal blood flow, and disruption of vasodilation through inactivation of vasodilator peptides.^40–45^ The specific role of suPAR in mediating adverse CVD outcomes and CKD progression needs further investigation. Similarly, the seemingly specific findings of KIM-1 with CRAE and CRVE require confirmation, as we did not observe associations with the other biomarkers of kidney tubule health.

Strengths of this study include measurement of multiple biomarkers reflecting distinct aspects of kidney tubule biology, including tubule injury, inflammation, and repair in a large, well-characterized population without CVD, CKD, or diabetes. With the exception of α1M, all biomarkers were measured in duplicate and averaged to improve precision, and we used standardized protocols to measure CRAE and CRVE. This study also has important limitations. First, the study included a generally healthy population excluding participants with underlying comorbidities including CVD, CKD, and diabetes. Our findings may have been different with the inclusion of participants with these diseases. The study had a cross-sectional design, precluding evaluation of temporal relationships between tubule health markers and retinal measures of microvascular disease. Finally, the study sample was relatively small, and retinal photography occurred 2 years after the urine tubule measurements on average. This temporal separation may have affected our interpretation of the observed associations. However, given the slow progression of subclinical disease and the overall health of the selected cohort, the effect of the time gap should be limited.^46,47^

In conclusion, among healthy community-living individuals selected for the absence of CVD, CKD, or diabetes and among six plasma and six urine biomarkers of kidney tubular health, higher levels of plasma and urine KIM-1 were associated with retinal arteriolar narrowing. However, among the other biomarkers, only one other (suPAR) was associated with retinal arteriolar diameter, and the direction was opposite to that observed with KIM-1. All other kidney tubule health biomarkers had null associations with the retinal vascular measures. These findings suggest that the link between kidney tubular health and retinal microvascular structure may reflect specific biologic pathways such as inflammation or endothelial injury rather than a broad manifestation of tubular dysfunction. Although this study was cross-sectional and focused on baseline associations, participants have since been followed longitudinally in MESA, and future work can evaluate whether these early microvascular and tubular alterations predict long-term cardiovascular or kidney outcomes. Future studies are required to evaluate the link between kidney tubule health markers and microvascular disease and should broaden the assessment of tubule health markers, evaluate microvascular disease in other vascular beds, and link findings with longitudinal CVD events.

## Supplementary Material

SUPPLEMENTARY MATERIAL

## Data Availability

This study includes clinical experimentation and received Institutional Review Board or Ethics Committee approval. All patients provided written informed consent.

## Data Availability Statement

Original data cannot be shared. The data that support the findings of this study are available from the MESA Coordinating Center. The authors are restricted from further distribution of the data. Interested investigators can request and access MESA deidentified datasets by completing a Data Distribution Agreement that will be reviewed for approval by the MESA Publications and Steering Committees. The data request is accessible *via* the following website: https://biolincc.nhlbi.nih.gov/studies/mesa.

## Supplemental Material

Supplemental Table 1. Plasma and urine kidney tubule biomarker concentrations of study participants (*n*=470).
