# Supplementary material for: Associations of Biomarkers of Kidney Tubule Health with Retinal Microvascular Signs: The Multi-Ethnic Study of Atherosclerosis
Source: Kidney360. 2025 Sep 5;6(12):2157–65. doi: 10.34067/KID.0000000970 (PMC12708413; doi:10.34067/KID.0000000970)
Supplement: SUPPLEMENTARY MATERIAL [file kidney360-6-2157-s001.pdf]

ASN
American Society of Nephrology
JASN
Journal of the American Society of Nephrology
KIDNEY360
Accessing Our World From Every Angle
CJASN
Clinical Journal of the American Society of Nephrology

ASN Journal Disclosure Form

As per ASN journal policy, I have disclosed any financial relationships or commitments I have held in the past 36 months as included below. I have listed my Current Employer below to indicate there is a relationship requiring disclosure. If no relationship exists, my Current Employer is not listed.

A. Ahmadi reports the following:
Employer: University of California San Diego

I understand that the information above will be published within the journal article, if accepted, and that failure to comply and/or to accurately and completely report the potential financial conflicts of interest could lead to the following: 1) Prior to publication, article rejection, or 2) Post-publication, sanctions ranging from, but not limited to, issuing a correction, reporting the inaccurate information to the authors' institution, banning authors from submitting work to ASN journals for varying lengths of time, and/or retraction of the published work.

Name: Armin Ahmadi
Manuscript ID: K360-2025-000546R1
Manuscript Title: Associations of Biomarkers of Kidney Tubule Health with Retinal Microvascular Signs: The Multi-Ethnic Study of Atherosclerosis (MESA)
Date of Completion: July 16, 2025
Disclosure Updated Date: May 12, 2025

ASN American Society of Nephrology
JASN Journal of the American Society of Nephrology
KIDNEY360 Accessing Our World From Every Angle
CJASN Clinical Journal of the American Society of Nephrology

ASN Journal Disclosure Form

Date: July 18, 2025

Author: Matthew Allison

Manuscript ID: K360-2025-000546R1

Manuscript Title: Associations of Biomarkers of Kidney Tubule Health with Retinal Microvascular Signs: The Multi-Ethnic Study of Atherosclerosis (MESA)

Disclosure Statements

I have no financial disclosures for this report.

ASN American Society of Nephrology | JASN Journal of the American Society of Nephrology | KIDNEY360 Accessing Our World From Every Angle | CJASN Clinical Journal of the American Society of Nephrology

ASN Journal Disclosure Form

As per ASN journal policy, I have disclosed any financial relationships or commitments I have held in the past 36 months as included below. I have listed my Current Employer below to indicate there is a relationship requiring disclosure. If no relationship exists, my Current Employer is not listed.

M. Cotch reports the following:
Employer: National Institutes of Health

I understand that the information above will be published within the journal article, if accepted, and that failure to comply and/or to accurately and completely report the potential financial conflicts of interest could lead to the following: 1) Prior to publication, article rejection, or 2) Post-publication, sanctions ranging from, but not limited to, issuing a correction, reporting the inaccurate information to the authors' institution, banning authors from submitting work to ASN journals for varying lengths of time, and/or retraction of the published work.

Name: Mary Frances Cotch
Manuscript ID: K-360-2025-00546R1
Manuscript Title: Associations of Biomarkers of Kidney Tubule Health with Retinal Microvascular Signs: The Multi-Ethnic Study of Atherosclerosis (MESA)
Date of Completion: July 22, 2025
Disclosure Updated Date: July 22, 2025

ASN
American Society of Nephrology
JASN
Journal of the American Society of Nephrology
KIDNEY360
Accessing Our World From Every Angle
CJASN
Clinical Journal of the American Society of Nephrology

ASN Journal Disclosure Form

As per ASN journal policy, I have disclosed any financial relationships or commitments I have held in the past 36 months as included below. I have listed my Current Employer below to indicate there is a relationship requiring disclosure. If no relationship exists, my Current Employer is not listed.

H. Gorji reports the following:
Employer: Renal Research Institute; University of California in San Francisco

I understand that the information above will be published within the journal article, if accepted, and that failure to comply and/or to accurately and completely report the potential financial conflicts of interest could lead to the following: 1) Prior to publication, article rejection, or 2) Post-publication, sanctions ranging from, but not limited to, issuing a correction, reporting the inaccurate information to the authors' institution, banning authors from submitting work to ASN journals for varying lengths of time, and/or retraction of the published work.

Name: Hassan Gorji
Manuscript ID: K360-2025-000546R1
Manuscript Title: Associations of Biomarkers of Kidney Tubule Health with Retinal Microvascular Signs: The Multi-Ethnic Study of Atherosclerosis (MESA)
Date of Completion: August 20, 2025
Disclosure Updated Date: August 20, 2025

ASN
American Society of Nephrology
JASN
Journal of the American Society of Nephrology
KIDNEY360
Accessing Our World From Every Angle
CJASN
Clinical Journal of the American Society of Nephrology

ASN Journal Disclosure Form

As per ASN journal policy, I have disclosed any financial relationships or commitments I have held in the past 36 months as included below. I have listed my Current Employer below to indicate there is a relationship requiring disclosure. If no relationship exists, my Current Employer is not listed.

O. Gutierrez reports the following:
Employer: UAB School of Medicine; and Honoraria: Akebia; Amgen; Ardelyx.

I understand that the information above will be published within the journal article, if accepted, and that failure to comply and/or to accurately and completely report the potential financial conflicts of interest could lead to the following: 1) Prior to publication, article rejection, or 2) Post-publication, sanctions ranging from, but not limited to, issuing a correction, reporting the inaccurate information to the authors' institution, banning authors from submitting work to ASN journals for varying lengths of time, and/or retraction of the published work.

Name: Orlando M. Gutierrez
Manuscript ID: K360-2025-000546R1
Manuscript Title: Associations of Biomarkers of Kidney Tubule Health with Retinal Microvascular Signs: The Multi-Ethnic Study of Atherosclerosis (MESA)
Date of Completion: July 17, 2025
Disclosure Updated Date: February 25, 2025

ASN American Society of Nephrology JASN Journal of the American Society of Nephrology KIDNEY360 Accessing Our World From Every Angle CJASN Clinical Journal of the American Society of Nephrology

ASN Journal Disclosure Form

As per ASN journal policy, I have disclosed any financial relationships or commitments I have held in the past 36 months as included below. I have listed my Current Employer below to indicate there is a relationship requiring disclosure. If no relationship exists, my Current Employer is not listed.

A. Houben reports the following:
Employer: maastricht university medical center; Advisory or Leadership Role: STIMULUS research consortium, European Union (non paid); and Other Interests or Relationships: Board member of European Society for Microcirculation and Netherlands Vascular Biology Organization.

I understand that the information above will be published within the journal article, if accepted, and that failure to comply and/or to accurately and completely report the potential financial conflicts of interest could lead to the following: 1) Prior to publication, article rejection, or 2) Post-publication, sanctions ranging from, but not limited to, issuing a correction, reporting the inaccurate information to the authors' institution, banning authors from submitting work to ASN journals for varying lengths of time, and/or retraction of the published work.

Name: Alfons Jhm Houben
Manuscript ID: K360-2025-000546R1
Manuscript Title: Associations of Biomarkers of Kidney Tubule Health with Retinal Microvascular Signs: The Multi-Ethnic Study of Atherosclerosis (MESA)
Date of Completion: July 21, 2025
Disclosure Updated Date: July 21, 2025

ASN American Society of Nephrology | JASN Journal of the American Society of Nephrology | KIDNEY360 Accessing Our World From Every Angle | CJASN Clinical Journal of the American Society of Nephrology

ASN Journal Disclosure Form

As per ASN journal policy, I have disclosed any financial relationships or commitments I have held in the past 36 months as included below. I have listed my Current Employer below to indicate there is a relationship requiring disclosure. If no relationship exists, my Current Employer is not listed.

J. Ix reports the following:
Employer: UCSD; Consultancy: AstraZeneca, Bayer,; Research Funding: Breakthrough T1D; Advisory or Leadership Role: AlphaYoung; and Other Interests or Relationships: Executive Board for Kidney Disease: Improving Global Outcomes (KDIGO) -.

I understand that the information above will be published within the journal article, if accepted, and that failure to comply and/or to accurately and completely report the potential financial conflicts of interest could lead to the following: 1) Prior to publication, article rejection, or 2) Post-publication, sanctions ranging from, but not limited to, issuing a correction, reporting the inaccurate information to the authors' institution, banning authors from submitting work to ASN journals for varying lengths of time, and/or retraction of the published work.

Name: Joachim H. Ix
Manuscript ID: K360-2025-000546R1
Manuscript Title: Associations of Biomarkers of Kidney Tubule Health with Retinal Microvascular Signs: The Multi-Ethnic Study of Atherosclerosis (MESA)
Date of Completion: July 17, 2025
Disclosure Updated Date: July 17, 2025

ASN American Society of Nephrology JASN Journal of the American Society of Nephrology KIDNEY360 Accessing Our World From Every Angle CJASN Clinical Journal of the American Society of Nephrology

ASN Journal Disclosure Form

As per ASN journal policy, I have disclosed any financial relationships or commitments I have held in the past 36 months as included below. I have listed my Current Employer below to indicate there is a relationship requiring disclosure. If no relationship exists, my Current Employer is not listed.

R. Katz reports the following:
Employer: University of Washington

I understand that the information above will be published within the journal article, if accepted, and that failure to comply and/or to accurately and completely report the potential financial conflicts of interest could lead to the following: 1) Prior to publication, article rejection, or 2) Post-publication, sanctions ranging from, but not limited to, issuing a correction, reporting the inaccurate information to the authors' institution, banning authors from submitting work to ASN journals for varying lengths of time, and/or retraction of the published work.

Name: Ronit Katz
Manuscript ID: K360-2025-000546R1
Manuscript Title: Associations of Biomarkers of Kidney Tubule Health with Retinal Microvascular Signs: The Multi-Ethnic Study of Atherosclerosis (MESA)
Date of Completion: July 16, 2025
Disclosure Updated Date: July 16, 2025

ASN
American Society of Nephrology
JASN
Journal of the American Society of Nephrology
KIDNEY360
Accessing Our World From Every Angle
CJASN
Clinical Journal of the American Society of Nephrology

ASN Journal Disclosure Form

As per ASN journal policy, I have disclosed any financial relationships or commitments I have held in the past 36 months as included below. I have listed my Current Employer below to indicate there is a relationship requiring disclosure. If no relationship exists, my Current Employer is not listed.

B. Klein reports the following:
Employer: University of Wisconsin - Madison

I understand that the information above will be published within the journal article, if accepted, and that failure to comply and/or to accurately and completely report the potential financial conflicts of interest could lead to the following: 1) Prior to publication, article rejection, or 2) Post-publication, sanctions ranging from, but not limited to, issuing a correction, reporting the inaccurate information to the authors' institution, banning authors from submitting work to ASN journals for varying lengths of time, and/or retraction of the published work.

Name: Barbara E. K. Klein
Manuscript ID: K360-2025-000546R1
Manuscript Title: Associations of Biomarkers of Kidney Tubule Health with Retinal Microvascular Signs: The Multi-Ethnic Study of Atherosclerosis (MESA)
Date of Completion: July 17, 2025
Disclosure Updated Date: July 17, 2025

ASN American Society of Nephrology | JASN Journal of the American Society of Nephrology | KIDNEY360 Accessing Our World From Every Angle | CJASN Clinical Journal of the American Society of Nephrology

ASN Journal Disclosure Form

As per ASN journal policy, I have disclosed any financial relationships or commitments I have held in the past 36 months as included below. I have listed my Current Employer below to indicate there is a relationship requiring disclosure. If no relationship exists, my Current Employer is not listed.

R. Malhotra reports the following:
Employer: University of California San Diego

I understand that the information above will be published within the journal article, if accepted, and that failure to comply and/or to accurately and completely report the potential financial conflicts of interest could lead to the following: 1) Prior to publication, article rejection, or 2) Post-publication, sanctions ranging from, but not limited to, issuing a correction, reporting the inaccurate information to the authors' institution, banning authors from submitting work to ASN journals for varying lengths of time, and/or retraction of the published work.

Name: Rakesh Malhotra
Manuscript ID: K360-2025-000546R1
Manuscript Title: Associations of Biomarkers of Kidney Tubule Health with Retinal Microvascular Signs: The Multi-Ethnic Study of Atherosclerosis (MESA)
Date of Completion: July 17, 2025
Disclosure Updated Date: June 9, 2025

ASN American Society of Nephrology JASN Journal of the American Society of Nephrology KIDNEY360 Accessing Our World From Every Angle CJASN Clinical Journal of the American Society of Nephrology

ASN Journal Disclosure Form

As per ASN journal policy, I have disclosed any financial relationships or commitments I have held in the past 36 months as included below. I have listed my Current Employer below to indicate there is a relationship requiring disclosure. If no relationship exists, my Current Employer is not listed.

S. Meuer reports the following:
Employer: University of Wisconsin-Madison

I understand that the information above will be published within the journal article, if accepted, and that failure to comply and/or to accurately and completely report the potential financial conflicts of interest could lead to the following: 1) Prior to publication, article rejection, or 2) Post-publication, sanctions ranging from, but not limited to, issuing a correction, reporting the inaccurate information to the authors' institution, banning authors from submitting work to ASN journals for varying lengths of time, and/or retraction of the published work.

Name: Stacy M. Meuer
Manuscript ID: K360-2025-000546R1,
Manuscript Title: Associations of Biomarkers of Kidney Tubule Health with Retinal Microvascular Signs: The Multi-Ethnic Study of Atherosclerosis (MESA)
Date of Completion: July 17, 2025
Disclosure Updated Date: July 17, 2025

ASN American Society of Nephrology JASN Journal of the American Society of Nephrology KIDNEY360 Accessing Our World From Every Angle CJASN Clinical Journal of the American Society of Nephrology

ASN Journal Disclosure Form

As per ASN journal policy, I have disclosed any financial relationships or commitments I have held in the past 36 months as included below. I have listed my Current Employer below to indicate there is a relationship requiring disclosure. If no relationship exists, my Current Employer is not listed.

M. Sarnak reports the following:
Employer: My spouse works for Eli Lilly; Consultancy: Steering Committee of Trials Funded by Akebia; Ownership Interest: spouse is employee of Eli Lilly; and Research Funding: NIH.

I understand that the information above will be published within the journal article, if accepted, and that failure to comply and/or to accurately and completely report the potential financial conflicts of interest could lead to the following: 1) Prior to publication, article rejection, or 2) Post-publication, sanctions ranging from, but not limited to, issuing a correction, reporting the inaccurate information to the authors' institution, banning authors from submitting work to ASN journals for varying lengths of time, and/or retraction of the published work.

Name: Mark J. Sarnak
Manuscript ID: K360-2025-000546R1
Manuscript Title: Associations of Biomarkers of Kidney Tubule Health with Retinal Microvascular Signs: The Multi-Ethnic Study of Atherosclerosis (MESA)
Date of Completion: July 17, 2025
Disclosure Updated Date: February 26, 2025

ASN American Society of Nephrology JASN Journal of the American Society of Nephrology KIDNEY360 Accessing Our World From Every Angle CJASN Clinical Journal of the American Society of Nephrology

ASN Journal Disclosure Form

As per ASN journal policy, I have disclosed any financial relationships or commitments I have held in the past 36 months as included below. I have listed my Current Employer below to indicate there is a relationship requiring disclosure. If no relationship exists, my Current Employer is not listed.

S. Sashikanth has nothing to disclose.

I understand that the information above will be published within the journal article, if accepted, and that failure to comply and/or to accurately and completely report the potential financial conflicts of interest could lead to the following: 1) Prior to publication, article rejection, or 2) Post-publication, sanctions ranging from, but not limited to, issuing a correction, reporting the inaccurate information to the authors' institution, banning authors from submitting work to ASN journals for varying lengths of time, and/or retraction of the published work.

Name: Sanskrita Sashikanth
Manuscript ID: K360-2025-000546R1
Manuscript Title: Associations of Biomarkers of Kidney Tubule Health with Retinal Microvascular Signs: The Multi-Ethnic Study of Atherosclerosis (MESA)
Date of Completion: July 20, 2025
Disclosure Updated Date: July 20, 2025

ASN American Society of Nephrology | JASN Journal of the American Society of Nephrology | KIDNEY360 Accessing Our World From Every Angle | CJASN Clinical Journal of the American Society of Nephrology

ASN Journal Disclosure Form

As per ASN journal policy, I have disclosed any financial relationships or commitments I have held in the past 36 months as included below. I have listed my Current Employer below to indicate there is a relationship requiring disclosure. If no relationship exists, my Current Employer is not listed.

M. Shlipak reports the following:
Consultancy: University of Washington - CHS Cohort ; Bayer Health Pharmaceuticals; Research Funding: Bayer Pharmaceuticals; Advisory or Leadership Role: American Journal of Kidney Disease; Journal of the American Society of Nephrology; Circulation; Board Member and Chairman, Northern California Institute for Research and Education; and Other Interests or Relationships: Committee Member - KDIGO Guidelines Committee.

I understand that the information above will be published within the journal article, if accepted, and that failure to comply and/or to accurately and completely report the potential financial conflicts of interest could lead to the following: 1) Prior to publication, article rejection, or 2) Post-publication, sanctions ranging from, but not limited to, issuing a correction, reporting the inaccurate information to the authors' institution, banning authors from submitting work to ASN journals for varying lengths of time, and/or retraction of the published work.

Name: Michael Shlipak
Manuscript ID: K360-2025-000546R1
Manuscript Title: Associations of Biomarkers of Kidney Tubule Health with Retinal Microvascular Signs: The Multi-Ethnic Study of Atherosclerosis (MESA)
Date of Completion: July 17, 2025
Disclosure Updated Date: July 17, 2025

ASN American Society of Nephrology | JASN Journal of the American Society of Nephrology | KIDNEY360 Accessing Our World From Every Angle | CJASN Clinical Journal of the American Society of Nephrology

ASN Journal Disclosure Form

As per ASN journal policy, I have disclosed any financial relationships or commitments I have held in the past 36 months as included below. I have listed my Current Employer below to indicate there is a relationship requiring disclosure. If no relationship exists, my Current Employer is not listed.

R. Weinreb reports the following:
Consultancy: Abbvie, Alcon, Allergan, Amydis, Balance, Eyenovia, Iantrek, IOPtic, Implandata, Qlaris, Spinogenix, Toku,; Topcon.; Ownership Interest: Amydis, Eye-Go, Iantrek, MachineMD, Spinogenix, Toromedes, Toku.; Research Funding: Research instruments from iCare, Vasoptic, and Visionix; Founder and Ip licensed from UCSD for Toromedes; IP licenses from UCSD for Zeiss. Grants from National Institutes of Health, National Institute on Minority Health and Health Disparities, and Research to Prevent Blindness (New York, NY).; Patents or Royalties: Toromedes licensed by UCSD.; and Advisory or Leadership Role: On Board of Directors at Eye-Go, Iantrek, Toku and MachineMD.

I understand that the information above will be published within the journal article, if accepted, and that failure to comply and/or to accurately and completely report the potential financial conflicts of interest could lead to the following: 1) Prior to publication, article rejection, or 2) Post-publication, sanctions ranging from, but not limited to, issuing a correction, reporting the inaccurate information to the authors' institution, banning authors from submitting work to ASN journals for varying lengths of time, and/or retraction of the published work.

Name: Robert N. Weinreb
Manuscript ID: K360-2025-000546R1
Manuscript Title: Associations of Biomarkers of Kidney Tubule Health with Retinal Microvascular Signs: The Multi-Ethnic Study of Atherosclerosis (MESA)
Date of Completion: July 17, 2025
Disclosure Updated Date: July 17, 2025
