# Supplementary material for: Associations of Biomarkers of Kidney Tubule Health with Retinal Microvascular Signs: The Multi-Ethnic Study of Atherosclerosis
Source: Kidney360. 2025 Sep 5;6(12):2157–65. doi: 10.34067/KID.0000000970 (PMC12708413; doi:10.34067/KID.0000000970)
Supplement: SUPPLEMENTARY MATERIAL [file kidney360-6-2157-s002.pdf]

Supplement Table 1. Plasma and urine kidney tubule biomarker concentrations of study participants (n=470).

Plasma Biomarker	Median (IQR)	Log transformed (mean ± SD)
KIM-1 (pg/ml)	206 (140 to 320)	2.3 ± 0.3
MCP-1 (pg/ml)	127 (106 to 153)	2.1 ± 0.2
suPAR (pg/ml)	3085 (2488 to 3986)	3.5 ± 0.2
TNFR1 (pg/ml)	833 (649 to 1100)	2.9 ± 0.2
TNFR2 (pg/ml)	18364 (15385 to 22645)	4.3 ± 0.1
YKL-40 (pg/ml)	50510 (31303 to 92046)	4.7 ± 0.3
**Urine Biomarker**	**Median (IQR)**	**Log transformed (mean ± SD)**
AIM (mg/l)	5.6 (5.6 to 9.0)	0.9 ± 0.2
EGF (pg/ml)	7475 (5078 to 10617)	3.9 ± 0.3
KIM-1 (pg/ml)	1781.2 (991 to 2913)	3.2 ± 0.4
MCP-1 (pg/ml)	177 (104 to 290)	2.2± 0.4
YKL-40 (pg/ml)	376 (203 to 621)	2.6 ± 0.4
UMOD (pg/ml)	22624038 (14791358 to 35834582)	7.3 ± 0.3

Abbreviations: IQR, interquartile range; SD, standard deviation; KIM-1, kidney injury molecule-1; MCP-1, monocyte chemoattractant protein-1; suPAR, soluble urokinase-type plasminogen activator receptor; TNFR, tumor necrosis factor receptor; YKL-40, anti-chitinase-3-like protein 1; A1M, alpha-1-microglobulin; EGF, epidermal growth factor; A1M, alpha-1-microglobulin; UMOD, uromodulin.
